# Inhibitory effect of doxycycline conjugated with deoxycholic acid and polyethylenimine conjugate on nasal fibroblast differentiation and extracellular production

**DOI:** 10.1371/journal.pone.0285655

**Published:** 2024-05-16

**Authors:** Jae-Min Shin, Hyun-Woo Yang, Su-yeon Lim, Ji-hoon Jeong, Il-Ho Park

**Affiliations:** 1 Upper Airway Chronic Inflammatory Diseases Laboratory, Korea University College of Medicine, Seoul, South Korea; 2 Medical Device Usability Test Center, Guro Hospital, Korea University College of Medicine, Seoul, South Korea; 3 Department of Otorhinolaryngology-Head and Neck Surgery, Korea University College of Medicine, Seoul, South Korea; 4 School of Pharmacy, Sungkyunkwan University, Suwon, South Korea; IHRC, Inc. (Human Resource Service Administration), UNITED STATES

## Abstract

**Background:**

Chronic rhinosinusitis (CRS) is an inflammatory disease affecting the sinuses or nose. Persistent inflammatory responses can lead to tissue remodeling, which is a pathological characteristics of CRS. Activation of fibroblasts in the nasal mucosal stroma, differentiation and collagen deposition, and subepithelial fibrosis have been associated with CRS.

**Objectives:**

We aimed to assess the inhibitory effects of doxycycline and deoxycholic acid-polyethyleneimine conjugate (DA3-Doxy) on myofibroblast differentiation and extracellular matrix (ECM) production in nasal fibroblasts stimulated with TGF-β1.

**Methods:**

To enhance efficacy, we prepared DA3-Doxy using a conjugate of low-molecular-weight polyethyleneimine (PEI) (MW 1800) and deoxycholic acid (DA) and Doxy. The synthesis of the DA3-Doxy polymer was confirmed using nuclear magnetic resonance, and the critical micelle concentration required for cationic micelle formation through self-assembly was determined. Subsequently, the Doxy loading efficiency of DA3 was assessed. The cytotoxicity of Doxy, DA3, PEI, and DA-Doxy in nasal fibroblasts was evaluated using the WST-1 assay. The anti-tissue remodeling and anti-inflammatory effects of DA3-Doxy and DA3 were examined using real-time polymerase chain reaction (Real-time PCR), immunocytochemistry, western blot, and Sircol assay.

**Results:**

Both DA3 and DA3-Doxy exhibited cytotoxicity at 10 μg/ml in nasal fibroblasts. Doxy partially inhibited α-smooth muscle actin, collagen types I and III, and fibronectin. However, DA3-Doxy significantly inhibited α-SMA, collagen types I and III, and fibronectin at 5 μg/ml. DA3-Doxy also modulated TGF-β1-induced changes in the expression of MMP 1, 2, and 9. Nonetheless, TGF-β1-induced expression of MMP3 was further increased by DA3-Doxy. The expression of TIMP 1 and 2 was partially reduced with 5 μg/ml DA3-Doxy.

**Conclusions:**

Although initially developed for the delivery of genetic materials or drugs, DA3 exhibits inhibitory effects on myofibroblast differentiation and ECM production. Therefore, it holds therapeutic potential for CRS, and a synergistic effect can be expected when loaded with CRS treatment drugs.

## Introduction

Chronic rhinosinusitis (CRS) is a multifactorial inflammatory disease of the paranasal sinuses and nose. According to the European Guidelines, CRS persists for more than 12 weeks and is characterized by symptoms such as nasal congestion, anterior or posterior nasal drip, facial pain, and cough [1, 2]. CRS has various phenotypes depending on region, race, and environment, and it is generally classified into chronic rhinosinusitis with nasal polyp (CRSwNP) and chronic rhinosinusitis without nasal polyp (CRSsNP), depending on the presence or absence of nasal polyp. However, from a pathophysiological point of view, CRS is classified into Th1, Th2, and Th17 types [3, 4].

Although tissue remodeling is an important process for wound healing in the body, dysregulated tissue remodeling accompanied by ongoing inflammation can permanently change the histological composition of the tissue. This process induces irreversible changes in the tissue and promotes the continuous production and degradation of the extracellular matrix (ECM) [5]. Tissue remodeling is one of the pathological characteristics of CRS, involving phenomena such as fibroblast activation, myofibroblast differentiation, subepithelial fibrosis, and ECM production and degradation [6].

Transforming growth factor beta 1 (TGF-β1) is a cytokines related to cell division and differentiation. It stimulated the differentiation of fibroblasts into myofibroblasts and enhances their capacity to synthesize ECM proteins, including collagen, which leads to tissue remodeling, such as stromal fibrosis [7, 8]. Consequently, an in vitro experimental model involving fibroblast treatment with TGF-β1 can be used to investigate myofibroblast differentiation and ECM generation, both characteristic of fibrosis in tissue remodeling. Numerous studies have focused on inhibiting pathological tissue remodeling in chronic rhinosinusitis (CRS) or restoring deformed tissue to its original state [9, 10].

Doxycycline (Doxy) is a tetracycline antibiotic used for treating bacterial or parasitic infections and is commonly prescribed for chronic rhinosinusitis. Typically, Doxy is administered as a second-line treatment for patients experiencing corticosteroid side effects or for diabetic patients requiring long-term, low-dose therapy [11, 12]. *In vitro* studies have shown that Doxy can inhibit tissue remodeling and inflammation in nasal fibroblasts. To enhance its effectiveness, we conjugated deoxycholic acid (DA)-polyethylenimine (PEI) (DA3) with Doxy [13].

DA3, a construct synthesized from an amphiphilic bile acid and a non-toxic, low-molecular-weight PEI, facilitates the delivery of genetic materials or drugs into cells. In this study, we synthesized a DA3-Doxy conjugate and evaluated its inhibitory effects on myofibroblast differentiation and ECM production in nasal fibroblasts. Additionally, we assessed the anti-tissue remodeling potential of DA3 alone in the context of CRS.

## Materials and methods

### Materials

Polyethylenimine (PEI 1.8kDa, M_W_ 1800), deoxycholic acid (DA), dicyclohexyl carbodiimide (DCC), *N*-hydroxysuccinimide (NHS) were obtained from Sigma-Aldrich (St. Louis, MO, USA). Doxy was also purchased from Sigma-Aldrich.

### Synthesis of DA3 and DA3-Doxy conjugates

DA3 was synthesized following a previously described method [14]. Briefly, deoxycholic acid (DA, 2.5 mmol) was dissolved in tetrahydrofuran and activated using DCC/NHS chemistry.

The molar reaction stoichiometry of DA/DCC/NHS was 1:3:3. After 3-hour reaction at room temperature, activated DA was precipitated in n-hexane and dried under vacuum. The DA-PEI conjugate (DA3) was synthesized in dichloromethane by adding activated DA to PEI 1.8 KDa. The molar feed ratio of activated DA/PEI was 3:1. The reaction proceeded for 12 hours at room temperature. The solvent was then evaporated using a rotary evaporator, and the product was dissolved in 0.1 M hydrochloric acid before being precipitated in cold acetone/ether (1:3, v/v).

The mixture was centrifuged, the supernatant discarded, and the precipitate dried under dry nitrogen. The final product was dissolved in deionized water, filtered, and freeze-dried. The degree of substitution was determined by using ^1^H NMR (Methanol-d_4_): NMR (methanol-d_4_): δ = 1.0–2.5 (coprostane), 2.4–3.5 (CH_2_CH_2_N, 4H) (S1 Fig).

### Preparation of doxycycline-loaded DA3 micelles (DA3-Doxy)

DA3-Doxy micelles were prepared using a film casting and hydration method. One hundred milligrams of DA3 and 10 mg Doxy were dissolved in methanol. The solvent was removed using a rotary evaporator under reduced pressure to form a thin film. The film was then hydrated in deionized water and sonicated to obtain a homogenous suspension of DA3-Doxy. The resulting suspension was filtered (0.8 μm) and lyophilized. The drug loading content and efficiency of DA3-Doxy was analyzed using a fluorescence spectrophotometer.

### Human subjects

Normal uncinate process tissues (n = 3) were collected during rhinoplasty surgery. The subjects were recruited from the Department of Otorhinolaryngology at Korea University Medical Center, Korea. All tissues were obtained from patients without signs of inflammation, allergies, asthma, or aspirin sensitivity. Informed consent was acquired in accordance with the Declaration of Helsinki. This study was approved by the Korea University Medical Center Institutional Review Board and conducted following the guidelines of the Human Ethics Committee of the Korea University Guro Hospital (2020GR0308).

### Cell culture

Primary nasal fibroblasts were isolated from the uncinate process of patients who underwent rhinoplasty. To obtain the fibroblasts, uncinate process tissues were treated with enzymatic digestion with collagenase (500 U/mL; Sigma-Aldrich, St. Louis, MO), hyaluronidase (30 U/mL, Sigma-Aldrich), and DNase (10 U/mL, Sigma-Aldrich). Fibroblasts were cultured in Dulbecco’s Modified Eagle Medium (DMEM) containing 10% heat-inactivated fetal bovine serum (FBS) (Invitrogen, Carlsbad, CA), 10,000 μg/mL streptomycin (Invitrogen), and 1% 10,000 U/mL penicillin (Sigma-Aldrich). Passage 3–4 fibroblasts were used for *in vitro* experiments.

### WST-1 assay

To evaluate the cytotoxicity of DA3 components and Doxy, a WST-1 assay (DoGENBio, Korea) was performed. Primary nasal fibroblasts were seeded in a 96-well plate and treated with Doxy, PEI, DA, DA3, and DA3-Doxy for 24, 48, and 72 hours, respectively. Following treatment, 10 μL of WST-1 reagent was added to each well. The cells were incubated for 4 hours, and the results were measured using a spectrophotometer at a wavelength of 450 nM. To exclude the effect of proliferation, fibroblasts were treated with mitomycin C (5 μg/mL).

### Real-time polymerase chain reaction

To assess the RNA levels, we performed Real-time PCR. Nasal fibroblasts were seeded into 35-60mm culture dishes. After the cells reached 70–80% confluency, the respective treatments were applied. Total RNA was extracted from the treated fibroblasts using the Trizol reagent (Invitrogen). The extracted RNA was then quantified using a NanoDrop spectrophotometer. Next, 1 μg of total RNA was reverse-transcribed into cDNA using the Maxime RT PreMix kit (Intron Biotechnology, Korea), adhering to the manufacturer’s protocol. The real-time PCR amplification reactions were set up as follows: 10 μL of SYBR Green PCR Master Mix, 1 μL of each primer (Information on primers is shown in Table 1), 1 μL of the diluted cDNA template, and nuclease-free water to reach a total volume of 20 μL. The amplification reactions were performed using a QuantStudio 3 system (Applied Biosystems, Foster City, CA) under the following conditions: an initial 2-minute denaturation step at 94°C; 40 cycles of 94°C for 5 seconds (denaturation), 60°C for 10 seconds (annealing), and 72°C for 20 seconds (extension). Relative gene expression analysis was conducted using the 2-ΔΔCT method, where the cycle threshold (CT) values of the target genes were normalized to the CT values of the internal control, GAPDH. The fold change in gene expression was calculated by comparing the normalized CT values between the treated and control groups.

**Table 1:** Sequences of real time-PCR oligonucleotide primers.

Primer		Sequence
*α-SMA*	**Forward** **Reverse**	**5’ –GTGTTGCCCCTGAAGAGCA-3’** **5’ – GCTGGGACATTGAAAGTCTCA-3’**
*Fibronectin*	**Forward** **Reverse**	**5’ –CGGTGGCTGTCAGTCAAAG-3’** **5’-AAACCTCGGCTTCCTCCATAA-3’**
*COL1A1*	**Forward** **Reverse**	**5’-GAGGGCCAAGACGAAGACATC-3’** **5’-CAGATCACGTCATCGCACAAC-3’**
*COL3A1*	**Forward** **Reverse**	**5’-GGAGCTGGCTACTTCTCGC-3’** **5’GGGAACATCCTCCTTCAACAG-3’**
*IL-6*	**Forward** **Reverse**	**5’-ACTCACCTCTTCAGAACGAATTG-3’** **5’-CCATCTTTGGAAGGTTCAGGTTG-3’**
*IL-8*	**Forward** **Reverse**	**5’-ACTGAGAGTGATTGAGAGTGGAC-3’** **5’-AACCCTCTGCACCCAGTTTTC-3’**
*GAPDH*	**Forward** **Reverse**	**5’-GGAGCGAGATCCCTCCAAAA-3’** **5’-GGCTGTTGTCATACTTCTCATG-3’**

### Chou-Talalay analysis

To investigate the potential synergistic effects between the components of the DA3-Doxy conjugate, a Chou-Talalay analysis was performed. Fibroblasts stimulated by TGF-β1 were exposed to DA3, Doxy, and DA3-Doxy to examine the suppressive impact on the mRNA expression of α-SMA, FN, and Col1A1. The target mRNA expression levels were charted as a function of concentration to create dose-effect curves. The Chou-Talalay method is a well-established technique for assessing the potential synergy, additivity, or antagonism between two or more substances. It involves determining the combination index (CI), a quantitative representation of the interaction between drugs. Synergy is indicated by a CI value less than 1, additivity by a CI value equal to 1, and antagonism by a CI value greater than 1. In this evaluation, fibroblasts were subjected to various concentrations of DA3, Doxy, and DA3-Doxy individually and in combination, after TGF-β1 stimulation. The mRNA levels of a-SMA, FN, and Col1A1 were assessed using real-time PCR. Dose-effect curves were subsequently produced using the mRNA expression data. GraphPad Prism 9 software was employed to plot the dose-effect curves and to compute the half-maximal inhibitory concentration (IC50) values for each treatment. The CI values were then calculated using the CompuSyn software in accordance with the manufacturer’s guidelines (S2 Fig).

### Western blot

Nasal fibroblasts were seeded into 60-mm culture dish at a density of 5 × 10^5^ cells/mL. The cells were lysed using RIPA buffer (Sigma-Aldrich) containing protease and phosphatase inhibitors (Sigma-Aldrich). Proteins samples were separated by 10% sodium dodecyl sulfate-polyacrylamide gel electrophoresis and transferred to polyvinyl difluoride membranes (Merck Millipore, Billerica, MA). The membranes were blocked with 5% skim milk. Blots were incubated with primary antibodies against fibronectin, GAPDH (Santa Cruz Biotechnology, Inc., Dallas, TX), α-smooth muscle actin (α-SMA; Millipore Inc., Billerica, MA), and collagen type I (Abcam, Cambridge, UK). The blots were visualized with HRP-conjugated secondary antibodies and an electrochemical luminescence system (Pierce, Rockford, IL). Images were analyzed using ImageJ software (NIH, Rockville, MD) and protein expression was normalized to GAPDH expression.

### Immunocytochemical staining

To evaluate protein expression and localization, immunocytochemical staining was performed. Before using the cell culture slides, they were coated with poly-L-lysine for 4 h, after which they were exposed to UV light for 6 h. Cells were seeded on the poly-L-lysine coated slides. Subsequently, the cells were fixed with 4% paraformaldehyde and permeabilized with phosphate-buffered saline containing 0.01% Triton X-100 for 10 min. Next, the cells were blocked with 3% bovine serum albumin for 1 h, incubated with primary antibodies; α-SMA (Millipore), Fibronectin (Santa Cruz Biotechnology, Inc), and collagen type 1 (Abcam), followed by incubation with secondary antibodies (Invitrogen). Finally, the cells were counterstained with 4’-6-diamidino-2-phenylindole for 10 min and the stained cells were visualized using a confocal laser scanning microscope (LSM700, Zeiss, Oberkochen, Germany).

### Enzyme-linked immunosorbent assay

The levels of IL-6 and IL-8 were measured using DuoSet ELISA kit (R&D Systems, Minneapolis, MN, USA). Fibroblasts (3 ×10^5^ cell) were seeded in 60-mm dish and treated with the drugs. Enzyme-linked immunosorbent assay was conducted using the supernatant of the cultured cells. Each well of a 96-well plate was coated overnight with antibodies. On the next day, the coated 96-well plate was blocked with blocking buffer for 1 h and washed using a wash buffer. Primary antibodies against IL-6 and IL-8 were added to the media, followed by incubation for 2 h. A substrate solution and stop solution were sequentially added, and the optical density of each well was determined within 30 min using a microplate reader.

### Statistical analysis

The results were obtained from at least three independent experiments. Statistical significance of differences between control and experimental data was analyzed using the unpaired t-test or one-way analysis of variance followed by Tukey’s test (GraphPad Prism, version 8, GraphPad Software, San Diego, CA). Significance was established at a 95% confidence level and p < 0.05 indicated statistical significance.

## Results

### Cytotoxic effect of PEI, DA3, Doxy and DA3-Doxy on nasal fibroblasts

**Fig 1:**
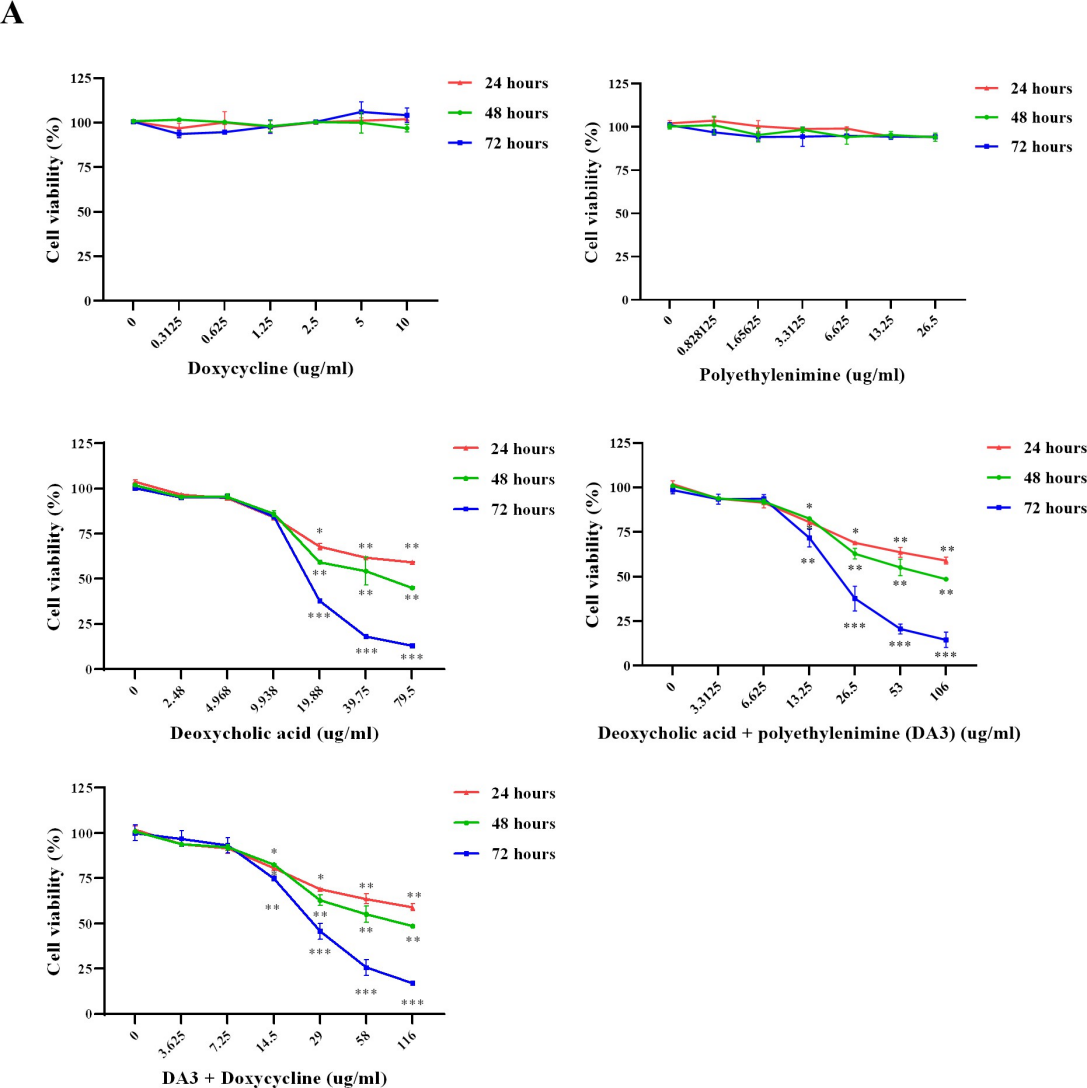
Assessment of cytotoxicity in nasal fibroblasts treated with DA3-Doxy components. (A) Doxy (0–10 μg/ml), PEI (0–26.5 μg/ml), DA (0–79.5 μg/ml), DA3 (0–106 μg/ml), and DA3-Doxy (0–116 μg/ml) were administered to nasal fibroblasts for 24, 48, or 72 hours. Cytotoxicity was evaluated using the WST-1 assay. Data from a minimum of three independent experiments are presented as mean ± standard deviation. One-way ANOVA was used to determine statistical significance. *p < 0.05, **p < 0.01, and ***p < 0.001, compared to non-treated fibroblasts. Abbreviations: Doxy, doxycycline; PEI, polyethylenimine; DA, deoxycholic acid; DA3, deoxycholic acid (DA)-polyethylenimine conjugate.

Cytotoxicity of Doxy, PEI, DA, DA3, and DA3-Doxy was evaluated in nasal fibroblasts. Considering the loading efficiency of Doxy in DA3, 116 μg/ml of DA3-Doxy was required to achieve a Doxy concentration of 10 μg/ml. DA3-Doxy is a mixture of DA, PEI, and Doxy, with DA and PEI combined at a ratio of 3:1. Excluding Doxy, the concentration of DA and PEI in 116 μg/ml of DA3-Doxy was 106 μg/ml, with 79.5 μg/ml of DA and 26.5 μg/ml of PEI. Cytotoxicity assessment was performed using the WST-1 assay, and nasal fibroblasts were treated for 24, 48, and 72 hours to evaluate toxicity. No cytotoxicity was observed in treatments with Doxy and PEI. However, cytotoxicity was observed in treatments containing DA, including DA, DA3, and DA3-Doxy. Cytotoxic effects were evident at concentrations of approximately 10 μg/ml and higher. However, in the fibroblast group treated with DA3, significant cytotoxicity was observed even at a concentration of 9.938 μg/ml (Fig 1). Therefore, we initially set the maximum concentration of DA3-Doxy for subsequent experiments at 5 μg/ml to minimize cytotoxic effects while assessing its therapeutic potential.

### Inhibition of TGF-β1-induced myofibroblast differentiation and ECM production by DA3 and DA3-Doxy in nasal fibroblasts

**Fig 2:**
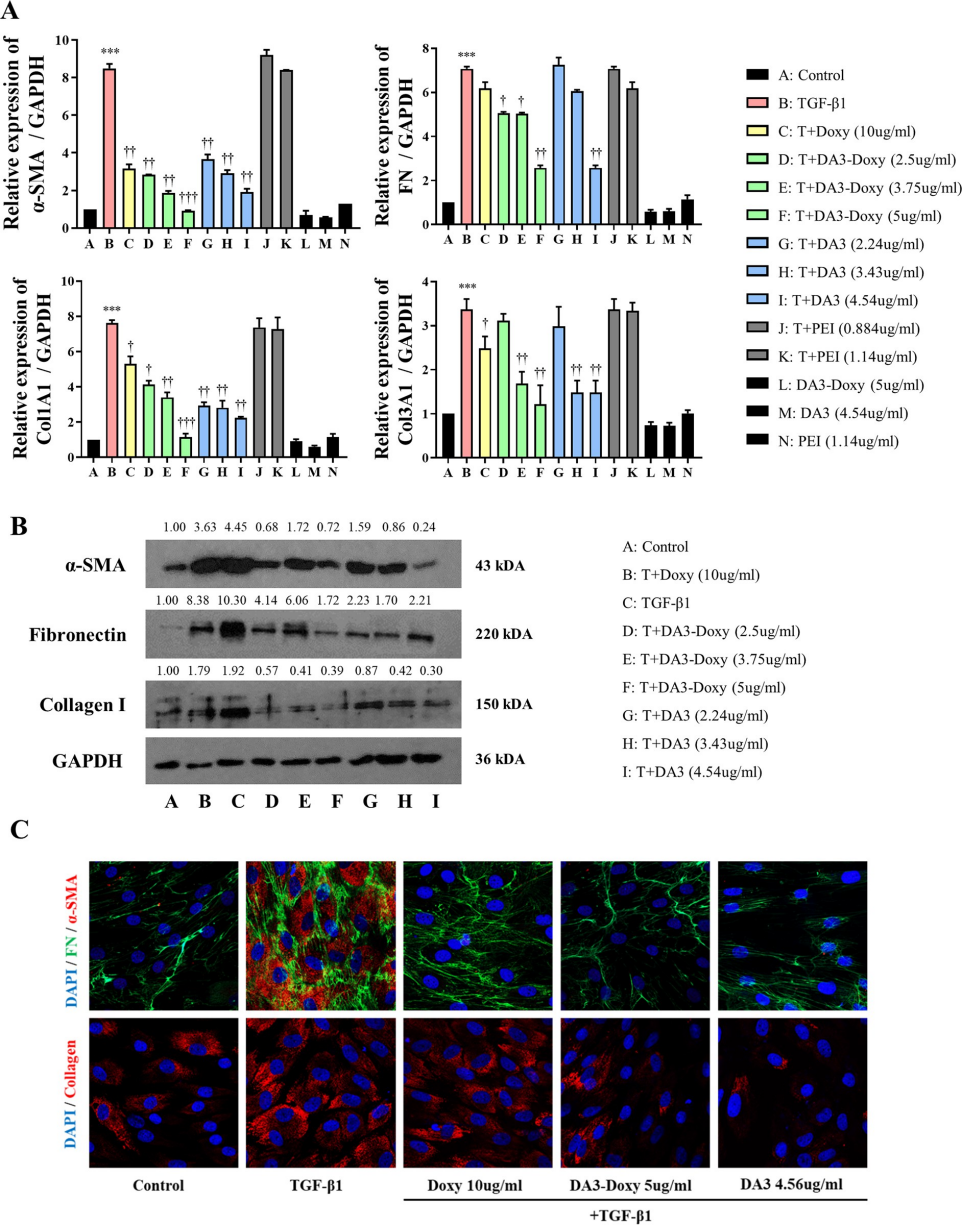
DA3 and DA3-Doxy attenuate TGF-β1-induced myofibroblast differentiation and collagen production. (A) Nasal fibroblasts were pre-treated with Doxy, DA3-Doxy, DA3 or PEI for 1 hour, followed by stimulation with TGF-β1 for 24, 48, and 72 hours. (B) mRNA levels of α-SMA were assessed after 24 hours of treatment. FN, Col1A1, and Col3A1 mRNA levels were assessed after 48 hours of stimulation using real-time PCR. (B) Protein expression levels were determined by western blot analysis after 72 hours of stimulation. (C) Immunofluorescent images of fibroblasts pre-treated with Doxy, DA3-Doxy or DA3 and stimulated with TGF-β1, or stimulated with TGF-β1 alone (red: α-SMA; upper panel, collagen type I; lower panel, green: fibronectin, blue: DAPI, ×400). Results were obtained from a minimum of three independent experiments. All results are presented as mean ± standard deviation. Statistical significance was determined using one-way analysis of variance tests. *p < 0.05 vs. untreated fibroblasts; **p < 0.01 vs. untreated fibroblasts; ***p < 0.001 vs. untreated fibroblasts. † p < 0.05 vs. TGF-β1-treated fibroblasts; ††p < 0.01 vs. TGF-β1-treated fibroblasts; †††p < 0.001 vs. TGF-β1-treated fibroblasts. Doxy, doxycycline; DA, deoxycholic acid; DA3, deoxycholic acid (DA)-polyethyleneimine copolymer; PEI, polyethyleneimine; TGF-β1, transforming growth factor-beta; α-SMA, alpha-smooth muscle actin; COL1A1, collagen type 1; COL3A1, collagen type 3; DAPI, 4’,6-diamidino-2-phenylindole.

To evaluate the inhibitory effects of Doxy, DA3, and DA3-Doxy on myofibroblast differentiation and extracellular matrix (ECM) production, we used nasal fibroblasts treated with TGF-β1 (5 ng/ml). TGF-β1 significantly increased the expression of α-SMA, COL1A1, COL3A1, and FN in nasal fibroblasts. The mRNA expression analysis revealed that Doxy decreased the expression of α-SMA, COL1A1, and COL3A1, but had minimal effect on FN. In contrast, both DA3 and DA3-Doxy reduced the expression of α-SMA, COL1A1, COL3A1, and FN in a dose-dependent manner. PEI, one of the components of DA3, did not exert any noticeable influence (Fig 2A). At the protein level, DA3 and DA3-Doxy reduced the expression of tissue remodeling-related factors, including α-SMA, COL1A1, and fibronectin, in a dose-dependent manner. Although Doxy did not significantly reduce FN at the mRNA level, it induced a meaningful reduction at the protein level (Fig 2B). To further investigate the expression and localization of related genes, immunocytochemistry (ICC) was performed TGF-β1 treatment increased the expression of α-SMA, COL1A1, COL3A1, and FN in the cytoplasm of nasal fibroblasts. Doxy effectively inhibited the TGF-β1-induced increase in α-SMA expression, while it only marginally suppressed FN. On the other hand, both DA3 and DA3-Doxy effectively inhibited the expression of α-SMA, COL1A1, and FN (Fig 2C).

### Regulation of MMP/TIMP family expression by DA3 and DA3-Doxy in nasal fibroblasts

**Fig 3:**
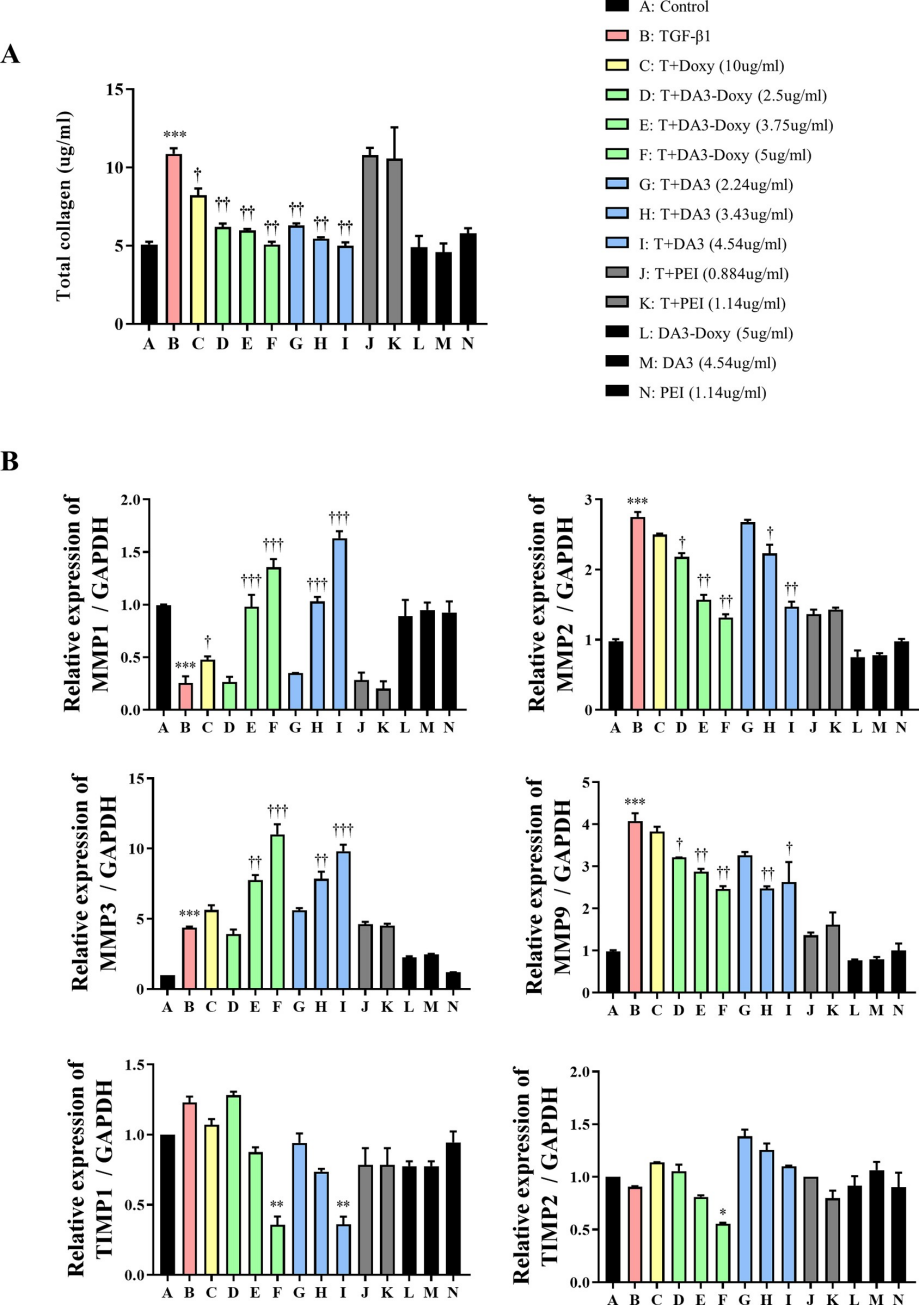
DA3 and DA3-Doxy regulate the expression of MMP/TIMP family in nasal fibroblasts. (A) Expression of total soluble collagen was evaluated using the Sircol assay. Nasal fibroblasts were pre-treated with Doxy, DA3-Doxy, DA3, or PEI for 1 hour and then stimulated with TGF-β1 for 72 hours. Subsequently, cell culture supernatants were collected, and Sircol reagent was applied to measure the amount of total collagen. (B) MMP1, 2, 3, 9, and TIMP1, 2 expression was assessed in nasal fibroblasts pre-treated with Doxy, DA3-Doxy, DA3, or PEI for 1 hour and then stimulated with TGF-β1 for 24 hours. Gene expression levels were determined by real-time PCR. Results were obtained from at least three independent experiments. All data are presented as mean ± standard deviation. Statistical significance was determined using one-way analysis of variance test. *p < 0.05 vs. untreated fibroblasts; **p < 0.01 vs. untreated fibroblasts; ***p < 0.001 vs. untreated fibroblasts. †p < 0.05 vs. TGF-β1-treated fibroblasts; ††p < 0.01 vs. TGF-β1-treated fibroblasts; †††p < 0.001 vs. TGF-β1-treated fibroblasts. Doxy, doxycycline; PEI, polyethyleneimine; DA, deoxycholic acid; DA3, deoxycholic acid (DA)-polyethyleneimine conjugate; TGF-β1, transforming growth factor-beta; MMP, matrix metalloproteinase; TIMP, tissue inhibitors of metalloproteinases.

Matrix metalloproteinases (MMPs) and tissue inhibitors of metalloproteinases (TIMPs) are enzymes responsible for the degradation of structural components of the extracellular matrix (ECM) in the body. MMPs are involved in several biological processes, including ECM hydrolysis, wound healing, tissue remodeling, and angiogenesis [15]. In CRS, irreversible tissue remodeling occurs through the repeated synthesis of ECM and degradation of interstitial tissue [16]. Therefore, we aimed to determine whether DA3 and DA3-Doxy could regulate the TGF-β1-induced changes in total collagen, MMPs, and TIMPs. To assess the collagen synthesis capability of nasal fibroblasts, we employed the Sircol assay. Doxy inhibited the TGF-β1-induced synthesis of total collagen. Both DA3-Doxy and DA3 further suppressed total collagen synthesis, exhibiting a more potent inhibition compared to Doxy, with a greater degree of inhibition as the concentration increased. PEI did not exert any influence on the TGF-β1-induced increase in total collagen (Fig 3A). TGF-β1 decreased the expression of MMP1, and Doxy reversed this decrease by increasing MMP1 expression. Both DA3-Doxy and DA3 elevated the mRNA levels of MMP1, even beyond that of the control group. While TGF-β1 significantly increased the expression of MMP2 and MMP9, Doxy did not exhibit inhibitory effects. In contrast, both DA3-Doxy and DA3 induced significant dose-dependent decreases in MMP2 and MMP9 expression. TGF-β1 upregulated MMP3 gene expression, and both DA3 and DA3-Doxy further enhanced MMP3 levels. Although TIMP1 and TIMP2 expression did not respond to TGF-β1 treatment, DA3-Doxy significantly inhibited their expression, while DA3 only modulated TIMP1 expression. PEI did not affect the MMP and TIMP family members (Fig 3B).

### DA3 does not inhibit TGF-β1-induced pro-inflammatory mediators

**Fig 4:**
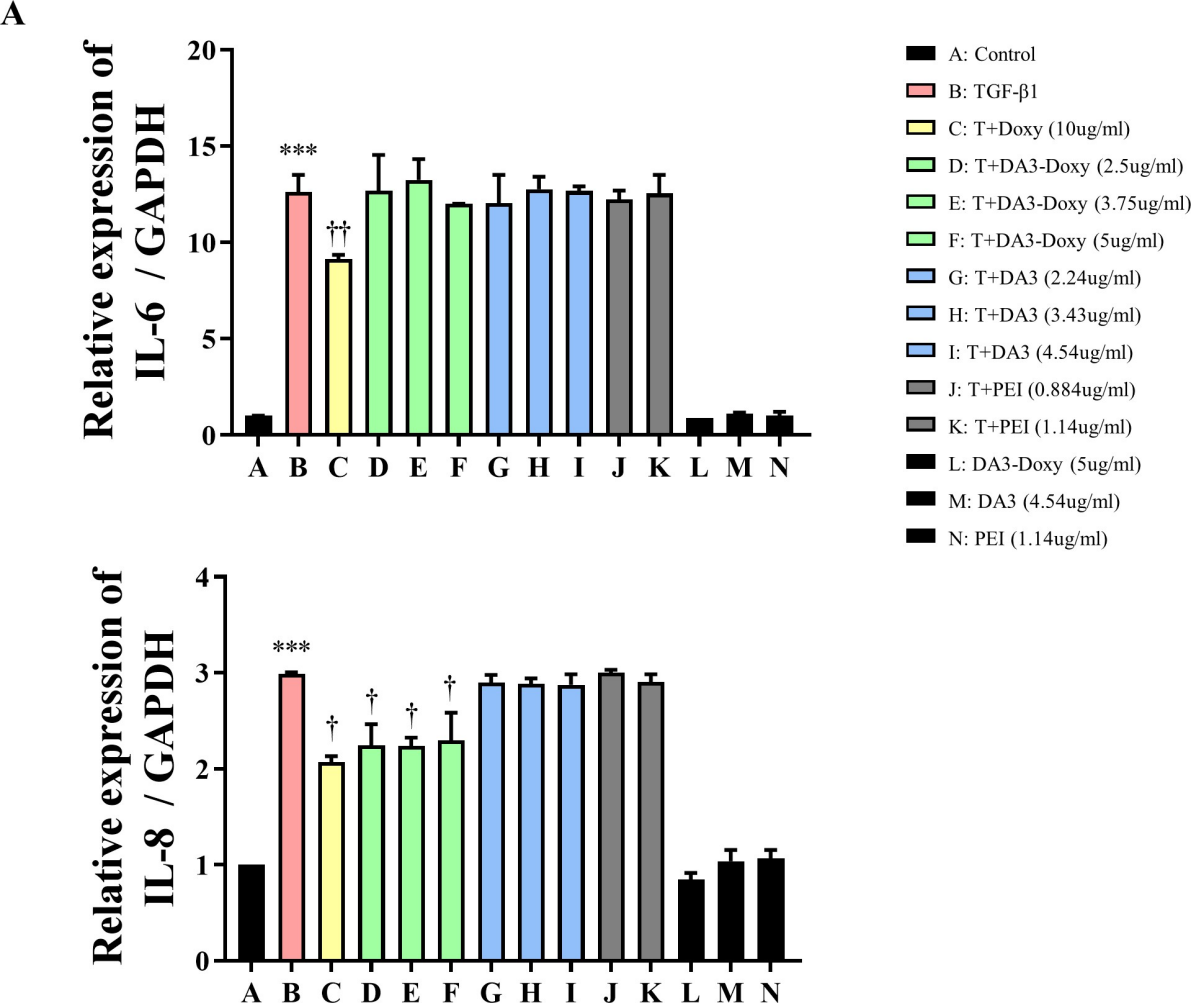
TGF-β1-induced pro-inflammatory mediators are not inhibited by DA3 and DA3-Doxy in nasal fibroblasts. Nasal fibroblasts were treated with Doxy, DA3, PEI, or DA3-Doxy for 1 hour and then stimulated with TGF-β1 for 48 hours. (A) IL-6 and IL-8 mRNA levels were assessed using real-time PCR. The results were obtained from at least three independent experiments. All results are expressed as mean ± standard deviation. Statistical significance was determined by one-way analysis of variance test. ***p < 0.001 vs. untreated fibroblasts. †p < 0.05 vs. TGF-β1-treated fibroblasts; ††p < 0.01 vs. TGF-β1-treated fibroblasts. Doxy, doxycycline; PEI, polyethyleneimine; DA, deoxycholic acid; DA3, deoxycholic acid (DA)-polyethyleneimine conjugate; TGF-β1, transforming growth factor-beta; IL-6, interleukin-6; IL-8, interleukin-8.

Persistent inflammatory responses can induce tissue remodeling. Inhibition of these inflammatory responses is one of the methods to control and suppress tissue remodeling [2, 17]. In an inflammatory environment, fibroblasts promote the secretion of IL-6 and IL-8. These IL-6 and IL-8 can facilitate the expression of cytokines and ECM in neighboring fibroblasts through autocrine or paracrine actions [18, 19]. Therefore, we evaluated the inhibitory effect of DA3 on pro-inflammatory mediators IL-6 and IL-8. TGF-β1 significantly increased the expression of IL-6 and IL-8 in nasal fibroblasts. Doxy at 10ug/ml significantly decreased the expression of IL-6; however, DA3-Doxy, DA3, and PEI failed to inhibit the TGF-β1-induced expression of IL-6. Both Doxy and DA3-Doxy partially reduced the TGF-β1-enhanced expression of IL-8, but DA3 and PEI did not show any effect (Fig 4A).

## Discussion

Fibroblasts play a critical role as structural cells within the stroma, responsible for the production of extracellular matrix (ECM) components, including collagen [20]. Under prolonged inflammatory conditions or exposure to specific cytokines such as transforming growth factor-β1 (TGF-β1), fibroblasts can differentiate into myofibroblasts. These myofibroblasts exhibit enhanced ECM production capacity compared to their fibroblast counterparts and can perpetuate tissue inflammation [21]. Within the stroma, myofibroblasts contribute to the formation of a fibrotic milieu by overexpressing ECM components and regulating the balance between matrix metalloproteinases (MMPs) and tissue inhibitors of metalloproteinases (TIMPs). This process results in significant tissue remodeling within the nasal mucosa and promotes the recruitment of immune cells. Additionally, myofibroblasts can stimulate angiogenesis by recruiting endothelial progenitor cells [22, 23]. Given the crucial role of myofibroblasts in the pathogenesis of CRS, strategies aimed at inhibiting myofibroblast differentiation and modulating ECM production could be promising therapeutic approaches to prevent pathological tissue remodeling in CRS and reduce disease recalcitrance.

DA3, a product formed by the reaction between the carboxyl group of bile acid and the amine group of polyethylenimine (PEI), has the potential to overcome a barrier in drug therapy. Generally, the limited permeability of DNA and proteins through the cell plasma membrane constrains the therapeutic efficacy of drugs. However, DA3 can address this issue due to its unique properties. In aqueous solutions, DA3 exhibits amphipathic behavior, spontaneously undergoing microphase separation to form self-assembled structures with a hydrophobic core and hydrophilic shell. This configuration allows for the loading and delivery of not only hydrophobic drugs but also anionic macromolecules or nanoparticles in the form of highly ionic complex nanoparticles. Owing to its ability to easily pass through the cell membrane, DA3 serves as an efficient carrier for genetic materials, proteins, and chemicals [24]. In our study, we conjugated DA3 with Doxy and investigated the effects of the resulting DA3-Doxy complex on various cellular processes.

The loading efficiency of Doxy in the DA3-Doxy conjugate, which was found to be approximately 8.57%. At a concentration of 116 μg/ml of DA3-Doxy, 10 μg/ml of Doxy was loaded. While Doxy and PEI displayed no cytotoxicity, both DA3 and DA3-Doxy containing DA exhibited cytotoxic effects at concentrations ≥ 10 μg/ml. Given the 8.57% loading efficiency of doxycycline, the maximum deliverable concentration of Doxy using DA3 was determined to be 0.857 μg/ml.

In this study, we evaluated the potential inhibition of myofibroblast differentiation and ECM production in nasal fibroblasts by treating TGF-β1-stimulated fibroblasts with 2.5–5 μg/ml of DA3-Doxy and DA3, and using 10 μg/ml of Doxy as a drug control group. Doxy suppressed the expression of α-SMA, Col1A1, and Col1A1. However, minimal effects were observed at the mRNA level of FN. DA3-Doxy and DA3 significantly inhibited the expression of α-SMA, Col1A1, Col3A1, and FN in a concentration-dependent manner. Despite the actual concentration of Doxy in 5μg/ml DA3-Doxy being 0.43 μg/ml, it further reduced the expression of α-SMA and Col1A1 compared to DA3-treated fibroblasts. To confirm the synergistic effect between DA3 and Doxy, we performed a Chou-Talalay analysis and observed a combination index (CI) value of less than 1 for α-SMA, Col1A1, and FN. This confirmed the presence of a synergistic effect between the two drugs, which was more prominent in the inhibition of α-SMA and Col1A1 expression. These findings suggest that the expression of these genes is primarily regulated by DA3, but there is also a synergistic effect with Doxy. Therefore, DA3 not only enhances the delivery of Doxy but also induces a synergistic effect in inhibiting myofibroblast differentiation and ECM production. These results imply that the DA3-Doxy conjugate may hold potential as a novel therapeutic approach for preventing pathological tissue remodeling and reducing disease recalcitrance in chronic rhinosinusitis. The observed synergy between DA3 and Doxy suggests that the combination could provide a more effective treatment strategy than either drug alone, by simultaneously targeting multiple aspects of tissue remodeling in chronic rhinosinusitis. The dual-action of the DA3-Doxy conjugate in enhancing Doxy delivery and inhibiting myofibroblast differentiation and ECM production may lead to improved therapeutic outcomes for patients suffering from this condition.

MMPs and TIMPs are essential enzymes responsible for the breakdown and synthesis of ECM in the body. Imbalances between MMPs and TIMPs can disrupt ECM homeostasis, leading to tissue remodeling [23, 25]. In nasal fibroblasts, TGF-β1 stimulation decreased MMP-1 expression while significantly increasing the expression of MMP-2, 3, and 9. Additionally, TIMP-1 and 2 expression remained relatively unaffected. MMP-2 (gelatinase-A) and MMP-9 (gelatinase-B) contribute to tissue remodeling in mucosa by degrading collagen, laminin, and gelatin, including types IV, V, and XI collagen. Elevated expression of MMP-2 and MMP-9 has been reported in patients with CRS. In our previous study, we showed that TGF-β1-stimulated fibroblasts induced MMP-2 and 9 expression. Both DA3 and DA3-Doxy significantly modulated TGF-β1-induced changes in MMP expression, with TIMP-1 and 2 expression decreasing only at 5 μg/ml. The TGF-β1-induced increase in MMP3 expression was further enhanced by DA3 and DA3-Doxy. MMP3 degrades proteoglycans, fibronectin, and laminin, including types II, III, IV, and IX collagen, and activates other MMP family members such as MMP-1 and MMP-9. Therefore, the increased expression of MMP3 by DA3 and DA3-Doxy may have influenced the modulation of the reduced MMP-1, but the exact nature of this effect remains unclear. Further studies are needed to investigate the interaction or ratio of the MMP family with the TIMP family.

Doxy has been found to inhibit TGF-β1-induced pro-inflammatory mediators such as IL-6 and IL-8. However, no effect of DA3 and DA3-Doxy was observed on IL-6. IL-8 induced by TGF-B1 was partially inhibited by DA3-Doxy. According to these results, DA3 itself has nothing to do with the mechanism of suppressing the inflammatory response. On the other hand, depending on the loaded drug, DA-Drug conjugate may have an inflammatory response. However, when considering the loading efficiency of DA3, the concentration and size of the drug to be loaded should be fully considered.

## Conclusions

DA3 is useful for the delivery of genetic materials, such as DNA, RNA, siRNA, as well as anionic macromolecules and nanoparticles, into cells. It has the advantage of being less toxic than lipofectamine, but considering the loading efficiency, the concentration of the drug that can be loaded may be limited. However, the results of this study showed that DA3 and DA3-Doxy regulated tissue remodeling by inhibiting the differentiation of fibroblasts into myofibroblasts and their ability to produce ECM. Therefore, by acting as a carrier of other therapeutic candidates of chronic rhinosinusitis, such as siRNA, chemicals, and antibiotics, it can exert an improved or synergistic effect.

## Supporting information

S1 Fig Synthesis of DA3-Doxy conjugate. (A) The carboxyl group of deoxycholic acid (DA) was activated in THF through N-hydroxysuccinimide/dicyclohexyl carbodiimide chemistry, precipitated in n-hexane, purified, and dried under reduced pressure. To synthesize the conjugate, after dissolving the activated bile acid derivative in methylenechloride, polyethylenimine (PEI, Mw = 1.8 KDa) was added in a molar ratio of 1: 3 (PEI: DA), and the mixture was incubated for 3 h at room temperature and filtered to obtain dicyclohexylurea as a by-product. Dicyclohexylurea was dried under reduced pressure using a rotary evaporator. The dried product was dissolved in 0.1M hydrochloric acid, followed by precipitation in acetone, purification, drying, and dissolution in distilled water. The solution was filtered, and the filtrate was freeze-dried to obtain the polymer conjugates. (B) The synthesis of the polymer conjugate was confirmed using proton nuclear magnetic resonance. (C) The critical micelle concentration (cmc) was determined for self-assembly of polymer conjugates through fluorescence spectrum analysis using pyrene as a fluorescent probe. The PEI-DA conjugate formed cationic micelles through self-assembly at ≥ 16.1 mg/L (cmc = 16.1 mg/L). (D) Doxy loading efficiency of DA3. Doxy, doxycycline; PEI, polyethylenimine; DA, deoxycholic acid; DA3, deoxycholic acid (DA)-polyethylenimine conjugate; TGF-β1, transforming growth factor beta.
(TIF)

S2 Fig Verification of synergistic effect of DA3 and Doxy using Chou-Talalay analysis. Nasal fibroblasts were pre-treated with DA3, Doxy, or DA3-Doxy for 1 hour and then stimulated with TGF-β1 for 24 or 48 hours. The expression of α-SMA was assessed after 24 hours of stimulation, while the expression of FN and Col1A1 was assessed after 48 hours. The concentration ranges for each treatment were as follows: DA3 (0–5 μg/ml), Doxy (0–20 μg/ml), and DA3-Doxy (0–5 μg/ml). The inhibitory effects of each treatment on target mRNA expression were examined across these concentration ranges, and dose-response curves were generated. Using Prism 9, we determined the IC50 values for each treatment. Subsequently, the Chou-Talalay analysis was performed using the respective IC50 values and the ratio of DA3 to Doxy (0.914:0.086). This analysis enabled us to calculate the Combination Index (CI) values and assess the synergistic effects of the treatments on the three target molecules. The CI values for all three targets were below 1, indicating the presence of synergistic effects. Furthermore, we observed greater synergistic effects for α-SMA and Col1A1 compared to fibronectin.
(TIF)

S1 Raw images (PDF)
