# Supplementary figures and images for: Inhibitory effect of doxycycline conjugated with deoxycholic acid and polyethylenimine conjugate on nasal fibroblast differentiation and extracellular production
Source: PLoS One. 2024 May 16;19(5):e0285655. doi: 10.1371/journal.pone.0285655 (PMC11098436; doi:10.1371/journal.pone.0285655)

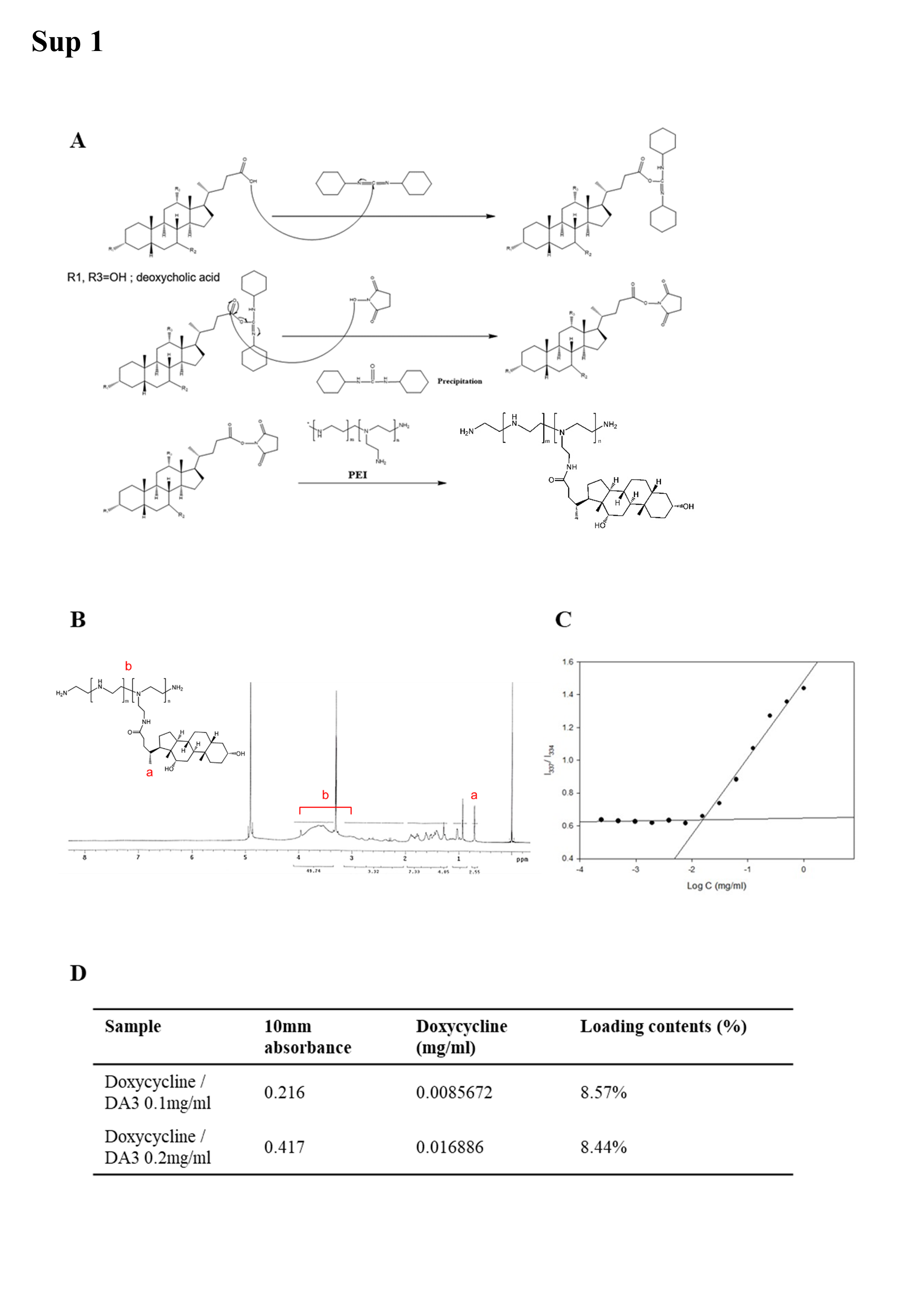

Supplement: S1 Fig — (A) The carboxyl group of deoxycholic acid (DA) was activated in THF through N-hydroxysuccinimide/dicyclohexyl carbodiimide chemistry, precipitated in n-hexane, purified, and dried under reduced pressure. To synthesize the conjugate, after dissolving the activated bile acid derivative in methylenechloride, polyethylenimine (PEI, Mw = 1.8 KDa) was added in a molar ratio of 1: 3 (PEI: DA), and the mixture was incubated for 3 h at room temperature and filtered to obtain dicyclohexylurea as a by-product. Dicyclohexylurea was dried under reduced pressure using a rotary evaporator. The dried product was dissolved in 0.1M hydrochloric acid, followed by precipitation in acetone, purification, drying, and dissolution in distilled water. The solution was filtered, and the filtrate was freeze-dried to obtain the polymer conjugates. (B) The synthesis of the polymer conjugate was confirmed using proton nuclear magnetic resonance. (C) The critical micelle concentration (cmc) was determined for self-assembly of polymer conjugates through fluorescence spectrum analysis using pyrene as a fluorescent probe. The PEI-DA conjugate formed cationic micelles through self-assembly at ≥ 16.1 mg/L (cmc = 16.1 mg/L). (D) Doxy loading efficiency of DA3. Doxy, doxycycline; PEI, polyethylenimine; DA, deoxycholic acid; DA3, deoxycholic acid (DA)-polyethylenimine conjugate; TGF-β1, transforming growth factor beta. (TIF) [file pone.0285655.s001.TIF]

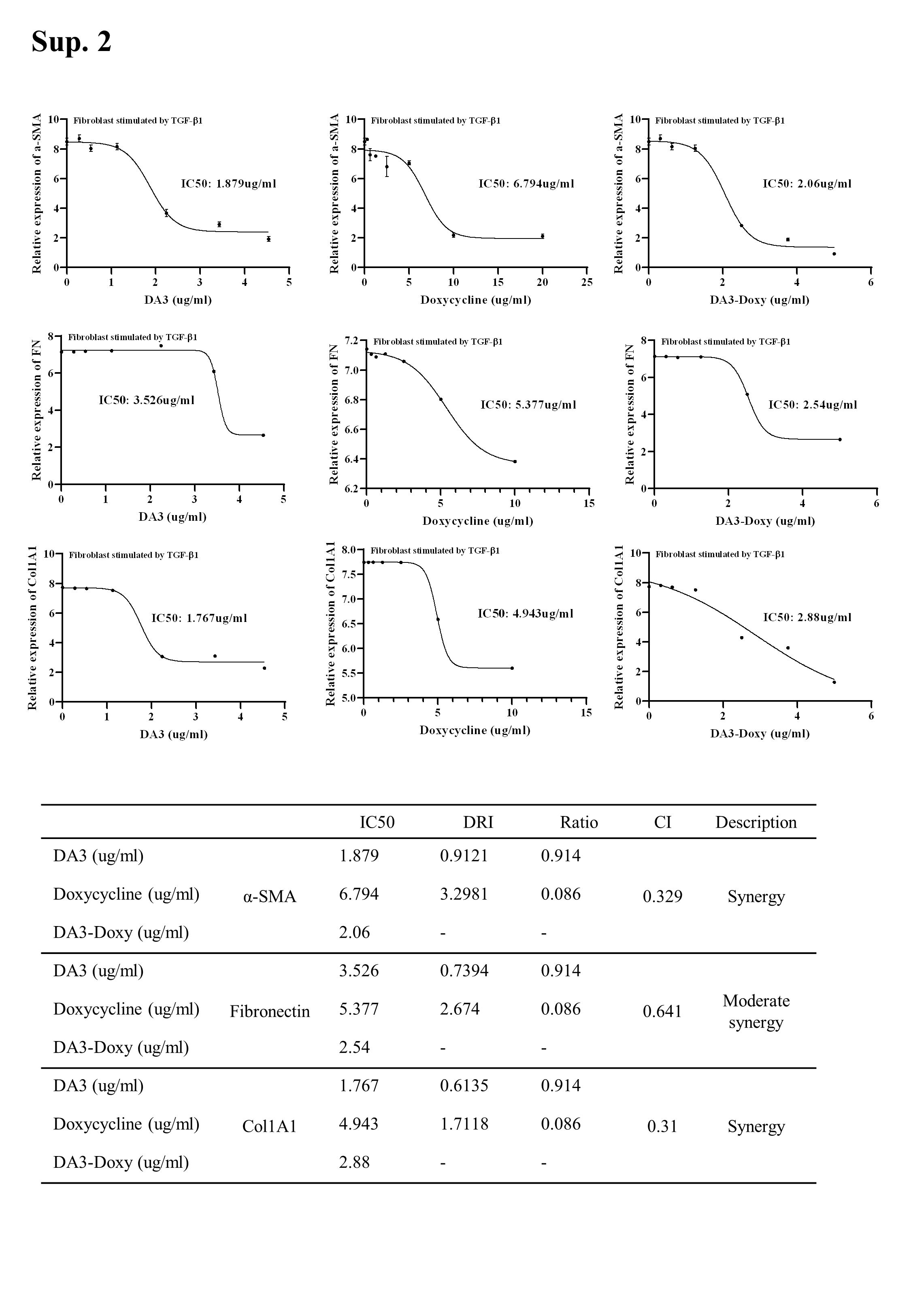

Supplement: S2 Fig — Nasal fibroblasts were pre-treated with DA3, Doxy, or DA3-Doxy for 1 hour and then stimulated with TGF-β1 for 24 or 48 hours. The expression of α-SMA was assessed after 24 hours of stimulation, while the expression of FN and Col1A1 was assessed after 48 hours. The concentration ranges for each treatment were as follows: DA3 (0–5 μg/ml), Doxy (0–20 μg/ml), and DA3-Doxy (0–5 μg/ml). The inhibitory effects of each treatment on target mRNA expression were examined across these concentration ranges, and dose-response curves were generated. Using Prism 9, we determined the IC50 values for each treatment. Subsequently, the Chou-Talalay analysis was performed using the respective IC50 values and the ratio of DA3 to Doxy (0.914:0.086). This analysis enabled us to calculate the Combination Index (CI) values and assess the synergistic effects of the treatments on the three target molecules. The CI values for all three targets were below 1, indicating the presence of synergistic effects. Furthermore, we observed greater synergistic effects for α-SMA and Col1A1 compared to fibronectin. (TIF) [file pone.0285655.s002.TIF]

S1\_raw\_images

$\alpha$ -SMA

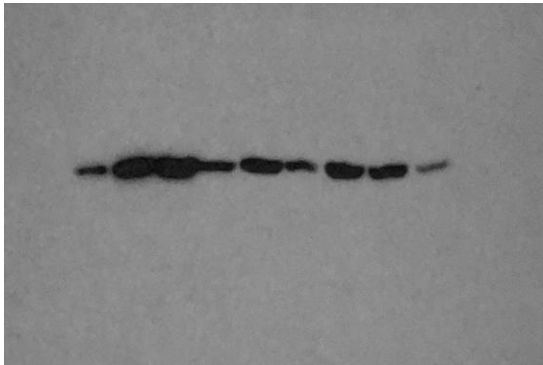

43KDa

Fibronectin

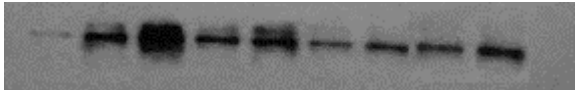

220KDa

Collagen type I

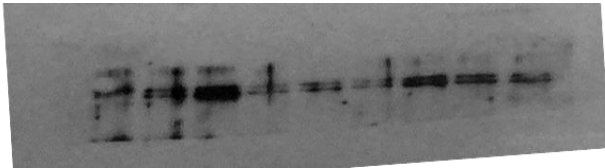

110KDa

GAPDH

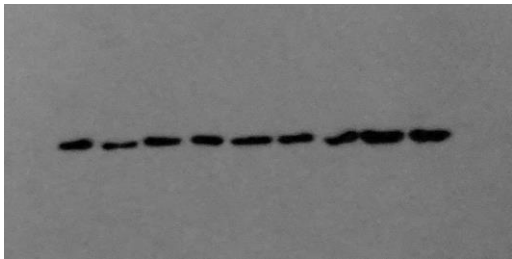

36KDa

Supplement: S1 Raw images — (PDF) [file pone.0285655.s003.pdf]
